# Capillary lactate concentration on admission of normotensive trauma patients: a prospective study

**DOI:** 10.1186/s13049-016-0272-x

**Published:** 2016-06-07

**Authors:** Pierre Bouzat, Clotilde Schilte, Marc Vinclair, Pauline Manhes, Julien Brun, Jean-Luc Bosson, Jean-François Payen

**Affiliations:** Pôle Anesthesie Reanimation, CHU Grenoble Alpes, F-38000 Grenoble, France; University Grenoble Alpes, F-38000 Grenoble, France; INSERM U1216, F-38000 Grenoble, France; Centre d’investigation clinique, CHU Grenoble Alpes, F-38000 Grenoble, France

**Keywords:** Lactate, Point-of-care, Transfusion, Severe trauma

## Abstract

**Background:**

Elevated serum blood lactate is an indicator of on-going bleeding in severe trauma patients. Point-of-care (POC) capillary lactate measurement devices may be useful to rapidly assess lactate concentration at the bedside. The aim of this study was to test the diagnostic performance of capillary lactate to predict significant transfusion in normotensive trauma patients.

**Methods:**

We conducted a prospective observational study in one level-I trauma centre. From August 2011 to February 2013, 120 consecutive adult patients with systolic blood pressure (SBP) higher than 90 mmHg were included. Capillary lactate was measured on admission in the trauma bay. The primary outcome was defined as a significant transfusion within the first 48 h. Diagnostic performance was determined using receiver operating characteristic (ROC) curve analysis. We also tested the agreement between capillary lactate and blood lactate concentrations using Bland and Altman analysis.

**Results:**

Of the 120 normotensive trauma patients, 30 (25 %) required at least one unit of packed red blood cells (RBC) and 12 (10 %) patients received at least four RBC within the first 48 h. All patients with significant RBC transfusion had capillary lactate higher than 3.5 mmol/l. The area under the ROC curve of capillary lactate on admission to predict transfusion of at least 4 RBC units was 0.68 [95 % CI 0.58 – 0.78]. The average bias between capillary and blood lactate measurements was 2.4 mmol/l with a standard deviation of 3.0 mmol/l (*n* = 60 patients).

**Conclusions:**

Although a significant association was found between POC lactate concentration and transfusion requirements, the diagnostic performance of capillary lactate measurements was poor. Due to large disagreement between capillary lactate and blood lactate, capillary lactate cannot be considered in the clinical setting.

**Trial registration:**

ClinicalTrials.gov, No. NCT01793428.

**Electronic supplementary material:**

The online version of this article (doi:10.1186/s13049-016-0272-x) contains supplementary material, which is available to authorized users.

## Background

Increase in lactate concentration is a common indicator of severity in critically ill patients [1–3]. After severe trauma, elevated serum lactate concentration was also associated with short-term outcomes. In preclinical studies, arterial lactate concentration was a strong predictor of blood loss after blunt or penetrating trauma [4–6]. In the clinical setting, initial lactate measurement was associated with organ failure and mortality in 129 trauma patients [7]. Blood lactate concentration was also an independent variable associated with mortality in 586 trauma patients [8]. Taken together, these studies highlight the role of arterial lactate concentration to screen high-risk patients for transfusion in the trauma bay.

Other classic vital parameters like heart rate or systolic arterial blood pressure (SBP) are common markers to predict critical bleeding in severe trauma patients [9]. Therefore, the additional information brought by serial lactate measurements may be obscured by classic physiological parameters in shocked patients. Interestingly, the yield of lactate concentration in a selected population of trauma patients with SBP between 90 and 110 mmHg allowed for early identification of patients requiring significant transfusion [10]. In patients with normal vital signs, elevated arterial lactate concentration was also found to be associated with occult major trauma [11]. These results were further corroborated in normotensive elderly blunt trauma patients [12]. Therefore, the additional benefit of arterial lactate concentration to detect patients at risk for transfusion may be superior in patients with normal vital signs compared to patients with shock on admission. However, measuring arterial lactate requires an automatic blood gas test and arterial blood sampling, which could be seen as invasive and expensive in patients with normal vital signs. To overcome these limitations, handheld point-of-care (POC) fingertip lactate measurement was implemented in emergency departments with adequate accuracy to determine moderate increase in lactate levels [13]. Using this device in the pre-hospital setting, capillary lactate concentration higher than 3.5 mmol/l was associated with in-hospital mortality in a study of 124 severe trauma patients requiring urgent ambulance dispatching [14].

The aim of the present study is to assess whether capillary lactate on admission can predict transfusion in trauma patients with a SBP higher than 90 mmHg. We also test the ability of the pre-hospital shock index defined as the ratio between heart rate and SBP to predict a significant transfusion. We assume that capillary lactate higher than 3.5 mmol/l can predict significant transfusion within the first 48 h post-trauma, with a higher accuracy than the pre-hospital shock index.

## Methods

### Study design and patients

We conducted a prospective observational study. Consecutive trauma patients with SBP higher than 90 mmHg were included from August 2011 to February 2013 in one level-I trauma centre (Grenoble University Hospital, Grenoble, France). The Regional Institutional Ethics Committee (Comité d’Ethique des Centres d’Investigation Clinique de l’inter-région Rhône-Alpes-Auvergne, IRB number 5708) approved the study design and, given its observational nature, waived the requirements for written informed consent from each patient. This study is registered with ClinicalTrials.gov No. NCT01793428.

Inclusion criteria were patients older than 18 year-old, admitted in the trauma bay for suspected severe trauma with a SBP higher than 90 mmHg. Severe trauma was suspected in the pre-hospital setting using the French Vittel triage criteria [15]. Exclusion criteria included pregnancy, chronic liver disease, pre-hospital transfusion, pre-hospital infusion of norepinephrine higher than 0.1 mcg/kg/min and body core temperature lower than 35 °C.

### Capillary lactate measurement

The handheld POC capillary lactate measurement device used in this study was the lactate scout® (Senslab, Leipzig, Germany). The lactate analyser is a small device with dimensions of 9.1 × 5.5 × 2.4 cm and weighing 85 g, including batteries. Reactive strips are used for the analysis of lactate using an enzymatic-amperometric biosensor as the measuring element. The measurement range goes from 0.5 to 25.0 mmol/l, and only 0.2 μl of blood is required for the analytical process.

### Study protocol and data collection

Patients were included at hospital admission, immediately after the pre-hospital phase. Two consecutive capillary lactate measurements were performed on admission concomitantly to capillary haemoglobin and glucose assessment. These measurements were performed by the nurse in charge of the patient using the same puncture. Sites of capillary puncture were located at fingertip or ear lobe. The average concentration of capillary lactate was recorded for analysis. Arterial or venous blood lactate concentration was concomitantly assessed when serum lactate concentration was prescribed by the attending physician. Arterial and venous lactate values were pooled since no discrepancy between these two variables was found in sepsis [16]. Physicians in charge of the patient were not informed about capillary lactate values.

The following clinical data was collected: age, gender, mechanism of injury, vital parameters (heart rate, SBP) in the pre-hospital field, pre-hospital shock index defined as the ratio between heart rate and SBP, total volume of infusion (colloid and crystalloids), number of RBC units transfused within the first 48 h, emergency treatment for haemostasis (embolization and/or damage control surgery including laparotomy, thoracotomy and orthopaedic surgery for haemostasis), length of stay in intensive care unit (ICU), injury severity score (ISS), and in-hospital mortality. Biological data consisted of capillary lactate and capillary haemoglobin. Serum blood lactate was also collected if available.

### Endpoints

The primary outcome was a significant transfusion within the first post-traumatic 48 h defined by a transfusion of at least six RBC units.

The secondary outcomes were: 1) blood lactate concentration to determine the agreement with the POC capillary lactate measurement; 2) a significant transfusion defined by at least four RBC units within the first 48 h; 3) the allocation of transfusion according to abnormal (≥3.5 mmol/l) or normal (< 3.5 mmol/l) capillary lactate concentration on admission.

### Study size

To be clinically relevant, we expected 90 % sensitivity for capillary lactate to predict a significant transfusion. The number of patients to be included was set at 120 patients to obtain an acceptable 95 % confidence interval (95 % CI) between 83 and 95 %.

### Statistical analysis

Descriptive statistics included frequencies and percentages for categorical variables, and median values (25^th^–75^th^ percentiles) for continuous variables. The diagnosic performance of capillary lactate to predict transfusion was evaluated using the area under of the receiver operating characteristic curve (AUC-ROC) with its 95 % confidence interval (95 % CI). Sensitivity and specificity were also calculated at the threshold that privileges sensitivity [17]. The agreement between the POC capillary lactate and the serum lactate concentration was done using a Bland & Altman representation [18]. Since only one measurement by each method was taken on each person, and the difference across the range was not constant, we decided to regress the differences on the averages and use the resulting equation to construct limits of agreement [19]. Comparisons between patients with capillary lactate higher or equal to 3.5 mmol/L and those with normal capillary lactate (< 3.5 mmol/l) were done using a chi-square test for categorical variables and using the Mann-Whitney non parametric test for continuous variables. Statistical analysis was performed with R software (version 3.1.2, https://cran.r-project.org). A *p* value of 0.05 or less was considered statistically significant.

## Results

We included 120 consecutive patients within the study period. Characteristics of the trauma population are summarized in Table 1. The typical patient was a young, male adult admitted for blunt trauma. Trauma severity in our cohort was moderate (median ISS = 19) and only two patients out of 120 (2 %) did not survive. Median concentrations of capillary and serum lactate are presented in Table 1. Thirty-two (27 %) patients required emergency treatment for haemostasis (embolization or damage control surgery). Seventy-five (63 %) patients were directed to the ICU after their admission into the trauma bay. Thirty (25 %) patients received at least one unit of RBC within the first 48 h. Only four (3 %) patients received a transfusion of at least six RBC units. The low incidence of the primary outcome did not allow us to explore the diagnostic performance of capillary lactate to predict transfusion of at least six RBC. Nevertheless, twelve (10 %) patients had at least four RBC units within the first 48 h (secondary outcome).

**Table 1:** Characteristics of the global population (*n* = 120 patients)

Variable	Value
Age, years	37 [27–56]
Male, *n* (%)	102 (85 %)
Blunt, *n* (%)	113 (94 %)
Prehospital SBP, mmHg	116 [100–131]
Prehospital HR, beats/min	80 [71–94]
Prehospital Shock index, *n* (%):	
< 0.9	81 (76 %)
> 0.9	26 (24 %)
Prehospital Crystalloids, ml	250 [0–500]
Prehospital Colloids, ml	0 [0–0]
Capillary lactate	3.7 [2.3–5.6]
Blood lactate*	1.6 [1.1–2.7]
Capillary hemoglobin, g/dl	13.6 [12.5–14.9]
Emergency surgery, *n* (%)	28 (23 %)
Embolization, *n* (%)	4 (3 %)
ISS	19 [10–26]
Length of stay in ICU, *n* (%)	7 [3–14]
Intra-hospital mortality, *n* (%)	2 (2 %)
Transfusion ≥ 4 RBC within 48 h, *n* (%)	12 (10 %)

Values are median [25^th^–75^th^ percentiles]

*RBC* packed red Blood Cell; *SBP* systolic blood pressure, *HR* heart rate, *ICU* intensive care unit, *ISS* injury severity score

*n = 60 patients

**Fig. 1:**
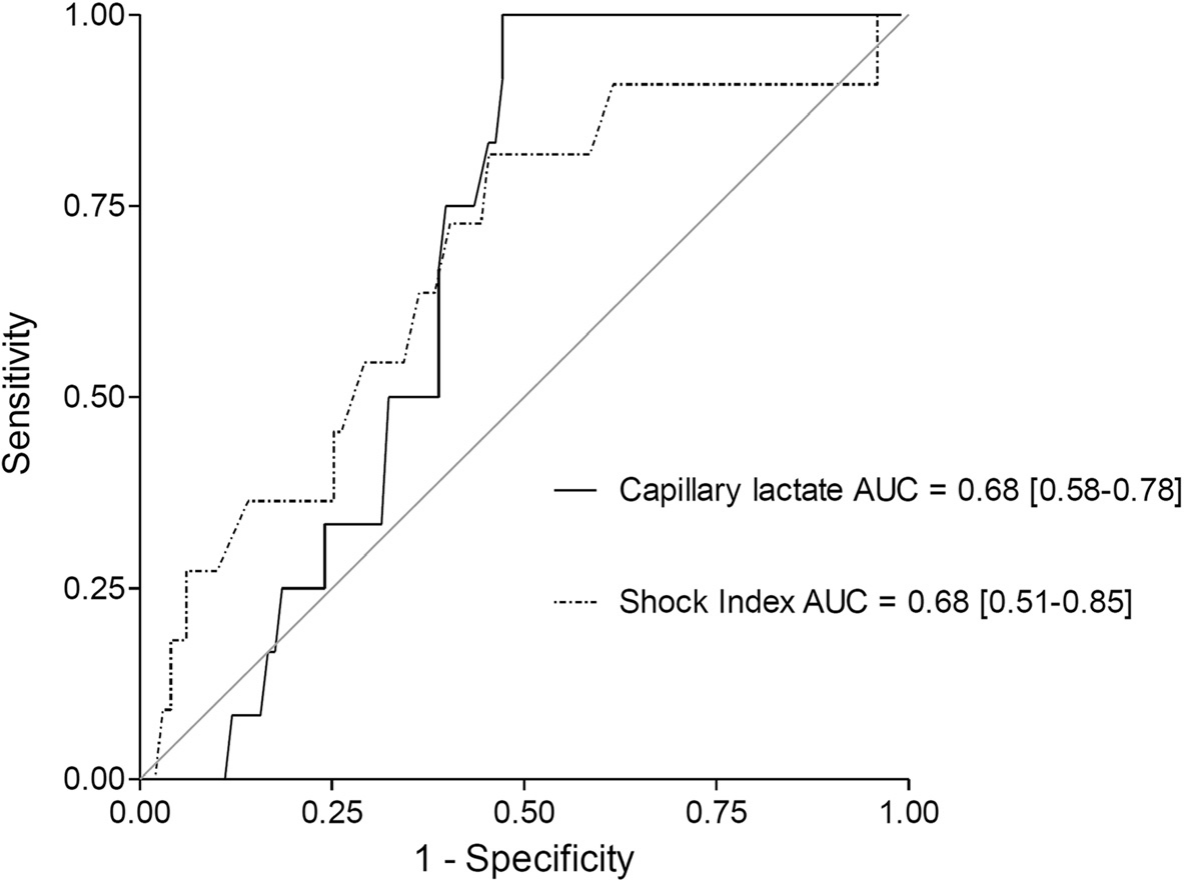
Receiver operating characteristic curves of capillary lactate on admission and prehospital shock index to predict a significant transfusion in the cohort (*n* = 120 patients)

The AUC-ROC of capillary lactate on admission to predict significant transfusion was 0.68 [95 % CI 0.58 – 0.78] (see Fig. 1 and Additional file 1). Maximizing the sensitivity, we found a threshold at 3.5 mmol/l. Sensitivity at this cut-off was 100 % (95 % CI 74–100 %) and specificity was 53 % (95 % CI 43–62 %). The AUC-ROC of the shock index was similar: 0.68 [95 % CI 0.51 – 0.85] (see Fig. 1 and Additional file 1). The AUC-ROC of capillary lactate on admission to predict any transfusion was only 0.59 [95 % CI 0.46 – 0.72] whereas the AUC-ROC of serum lactate was 0.77 [95 % CI 0.62 – 0.91].

**Fig. 2:**
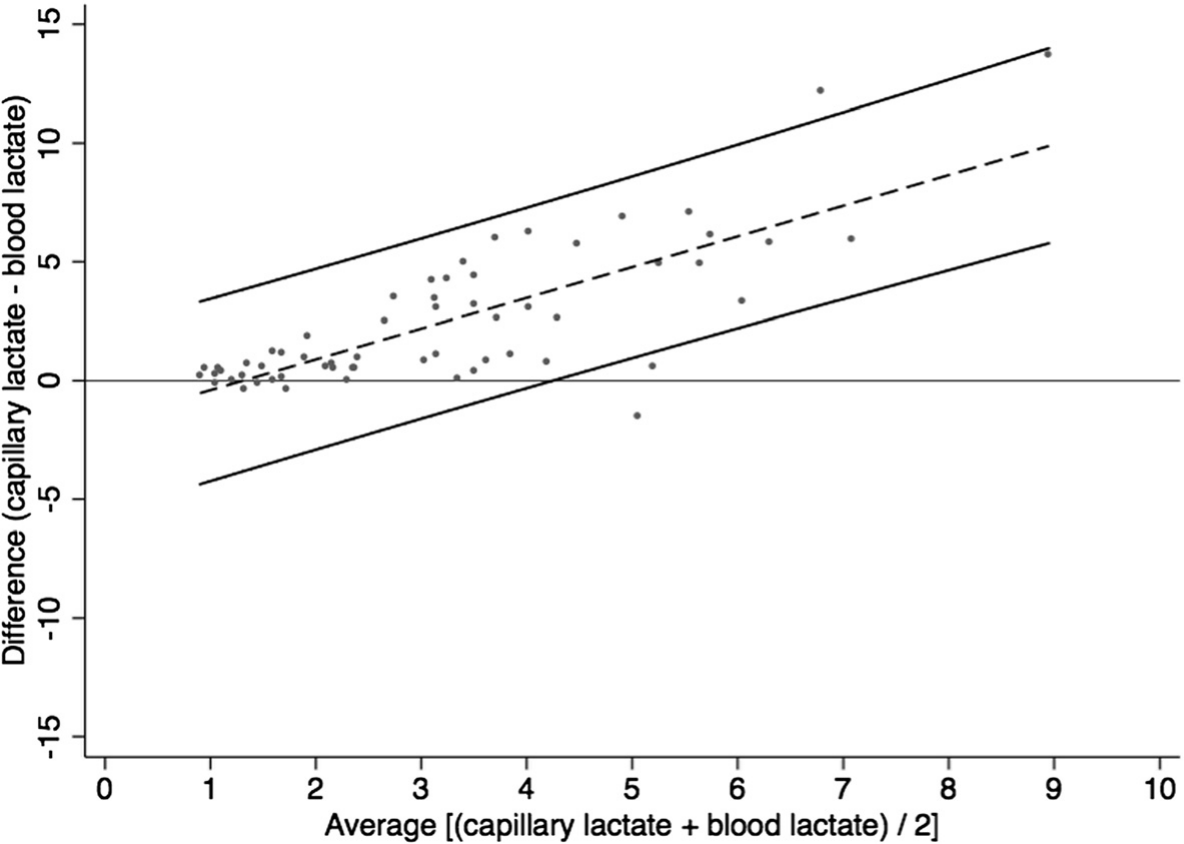
Agreement between the capillary lactate measurement device and serum blood lactate concentration via the Bland & Altman representation (*n* = 60 patients). Central dash-line represents the linear bias between the two methods. Upper and lower black lines represent the 95 % prediction limits for the blood lactate given the value by the capillary method

Agreement between capillary lactate and serum blood lactate was performed in 60 patients. Bland & Altman analysis is presented in Fig. 2. Average bias between the two methods was 2.4 mmol/l with a standard deviation of 3.0 mmol/l. The capillary lactate minus blood lactate difference was positively correlated with the capillary and blood lactate average (capillary – blood lactate = -1.6 + 1.26 × (capillary + blood lactate)/2, *p* < 0.001), indicating larger discrepancy between the two measurements in patients with abnormal lactate concentration. We used this linear bias to assess the 95 % prediction limits for the blood lactate given the value by the capillary method (Fig. 2).

**Fig. 3:**
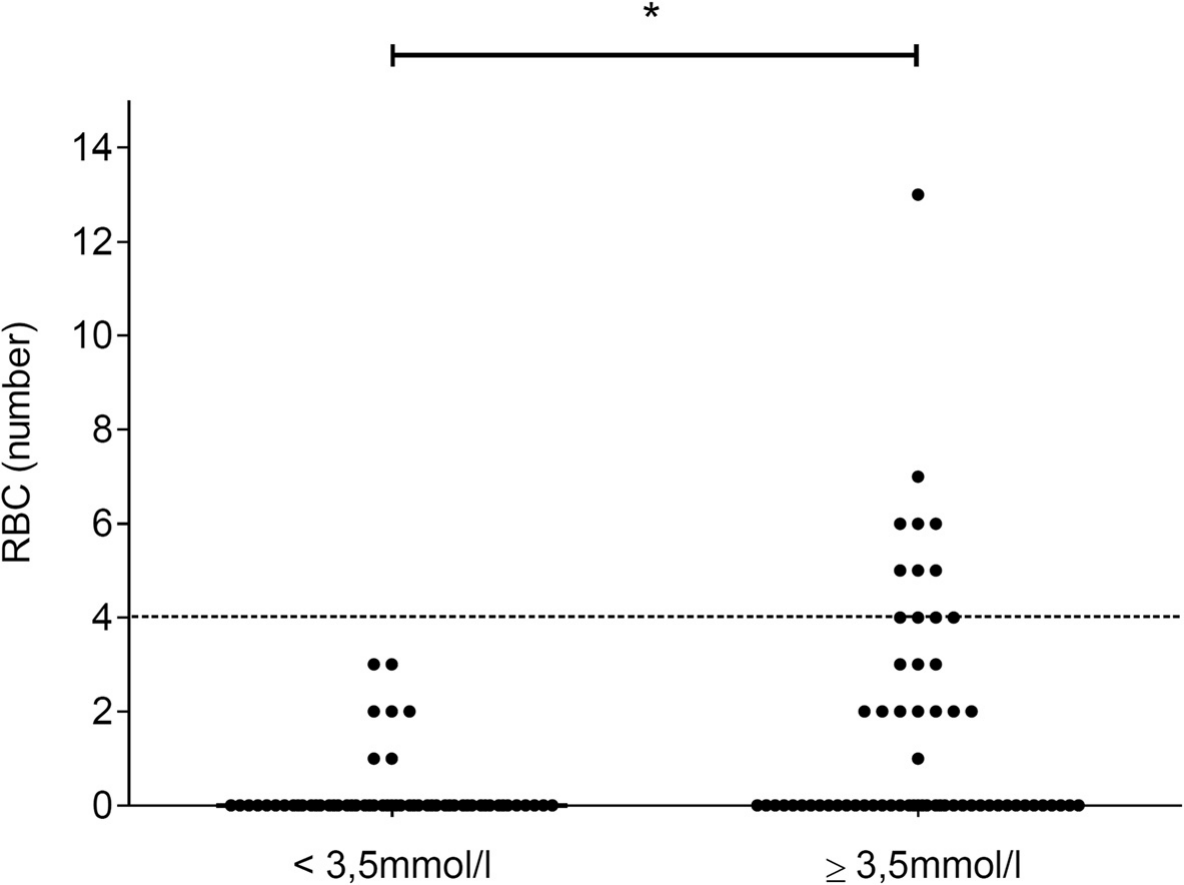
Allocation of packed red blood cells (RBC) units across individual patients according to the category of capillary lactate concentration on admission (< 3.5 mmol/l vs. ≥ 3.5 mmol/l). The dash-line represents the cut-off for significant transfusion (four RBCs). * *p* < 0.01

Univariate analyses between the group of patients with capillary lactate ≥ 3.5 mmol/l (*n* = 62 patients) and the group of patients with capillary lactate < 3.5 mmol/l (*n* = 58 patients) is shown on Table 2. Patients in the abnormal capillary lactate group had lower capillary haemoglobin, higher prehospital heart rate and more emergency haemostatic treatment than the normal capillary lactate group. The allocation of transfusion in the two groups is presented in Fig. 3. All patients receiving at least four RBC units had capillary lactate higher than 3.5 mmol/L.

**Table 2:** Univariate analysis between patients with capillary lactate < 3.5 mmol/l (*n* = 58 patients) versus patients with capillary lactate ≥ 3.5 mmol/l (*n* = 62 patients)

	Capillary lactate <3.5 mmol/l	Capillary lactate ≥3.5 mmol/l	*p*-value
	*N* = 58 patients	*N* = 62 patients	
Age, years	34.7 [26.3–55.1]	38 [28.2–56.5]	0.61
Male, *n* (%)	50 (86 %)	52 (84 %)	0.72
Blunt trauma, *n* (%)	51 (88 %)	60 (97 %)	0.47
Prehospital SBP, mmHg	120 [100–136]	111 [100–129]	
Prehospital HR, beats/min	77 [69–86]	82 [75–100]	0.26
Prehospital Shock index, *n* (%) :			0.9
< 0.9	42 (72 %)	42 (68 %)	**0.01**
> 0.9	16 (28 %)	20 (32 %)	
Prehospital Crystalloid, ml	250 [0–500]	250 [0–500]	
Prehospital Colloid, ml	0 [0–0]	0 [0–0]	
Capillary lactate	2.2 [1.4–2.6]	5.6 [4.5–7.2]	**<0.001**
Blood lactate*	1.45 [1–1.9]	2.1 [1.4–3.2]	**<0.01**
Capillary haemoglobin, g/dl	13.9 [13.0–15.0]	13.1 [11.7–14.5]	**0.02**
Emergency surgery, *n* (%)	10 (17.2 %)	18 (29 %)	0.13
Embolization, *n* (%)	0/58 (0 %)	4/62 (6.5 %)	**0.04**
ISS	19 [10–26]	18 [10–26]	0.9
Length of stay in ICU, *n* (%)	7 [3–19]	6.5 [3.3–12.8]	**<0.01**
In-hospital mortality, *n* (%)	1 (2 %)	1 (2 %)	0.58
Transfusion ≥4 RBC first 48 h, *n* (%)	0 (0 %)	12 (19 %)	0.96

Values are median [25th–75th percentiles]

*RBC* packed Red Blood Cell, *SBP* systolic blood pressure, *HR* heart rate, *ICU* intensive care unit, *ISS* injury severity score. *P* value < 0.05 are indicated in bold

*n = 60 patients

## Discussion

In a selected cohort of trauma normotensive patients, we found a poor diagnostic performance of capillary lactate to predict the transfusion of at least 4 RBC units. More concerning was the poor agreement between the capillary method and the serum blood lactate concentration. Although significant association was found between capillary lactate and transfusion requirements, the lack of accuracy of capillary lactate weakens the use of this POC device at the bedside to detect occult trauma in non-shocked trauma patients.

Capillary lactate higher than 3.5 mmol/l was statistically associated with transfusion requirements in our cohort. Indeed, this group of patients had more embolization procedures than patients with normal capillary lactate. Accordingly, transfusion needs were also higher in the capillary lactate ≥ 3.5 mmol/l group and all significantly transfused patients were part of this group. These findings were in line with prehospital evaluation of POC fingertip lactate device, where pre-hospital blood lactate levels were associated with in-hospital mortality [14]. In this study, capillary lactate was performed by nurses from the emergency medical service and provided more prognostic information than vital signs alone. In our study, we confirmed that capillary lactate could be achieved by the nurse in charge of the patient. However, we found statistical association but failed to demonstrate sufficient accuracy to predict transfusion needs. Indeed, the AUC-ROC was low with a lower limit of its 95 % confidence interval close to 0.5. This result clearly affected the diagnostic performance of capillary lactate at the early phase of severe trauma. In a larger retrospective cohort, blood lactate had larger AUC to predict significant transfusion at hospital admission (0.76) but no 95 % confidence interval was provided [10] and lactate was measured on blood samples. Interestingly, the same group found that prehospital shock index might facilitate the early identification of patients at risk for massive transfusion [20]. In our cohort of patients receiving less transfusion, we reported similar diagnostic performance for serum lactate (AUC ROC = 0.77 to predict any transfusion) but we did not report good diagnostic performance of prehospital shock index with an AUC-ROC equal to 0.68 [95 % CI 0.51 – 0.85]. Taken together, these findings illustrate the need for reliable information to detect occult severe trauma in normotensive patients. Unfortunately, in our study, neither capillary lactate nor shock index had sufficient diagnostic performance to be considered pertinent in determining patients’ severity at hospital admission.

The poor predictive value of capillary lactate might be related to the poor agreement between this method and the serum blood lactate measurement since average bias between the two methods was 2.4 mmol/l. Considering normal lactate range from 0 to 3.5 mmol/l, such bias limits the usefulness of POC device. One explanation for this result may be extra-capillary contamination by sweat composition [21]. Under intense physical exercise, lactate could be measured on the skin surface and may overestimate capillary lactate value. As severe trauma often occurs during sport-related accident in our region [22], this type of contamination may be expected in our patients. Other explanation may be interferences between capillary lactate assessment and interstitial oedema due to fluid overloading [23]. However, total infused volume was low in our study according to the moderate severity of the patients.

We acknowledge several limitations of our study. First, the incidence of the primary outcome was too low to explore capillary lactate in patients that received at least six RBC units. Nevertheless, the incidence of four RBC transfusions was relatively high in our cohort (10 %). Moreover, a transfusion of four RBC units may be considered significant from the clinical standpoint since this quantity represents a total volume of one litre. Second, the sample size of the cohort is relatively small and the limited number of events should be considered when interpreting our results. However the lack of agreement between the POC fingertip device and the reference method is valuable and questions the relevancy of capillary lactate measurement device in daily practice. Third, standardized capillary lactate measurement were not performed by one dedicated nurse. Nevertheless, this study was meant to be pragmatic and POC devices should be practical enough to be generalized in the trauma bay independently of the executing nurse.

## Conclusions

Capillary lactate was associated with transfusion requirements in normotensive trauma patients. However, we did not find sufficient accuracy of this technique for transfusion prediction at the bedside. The poor agreement between this device and the standard method may hinder the usefulness of capillary lactate in daily clinical practice. Despite encouraging statistical associations, capillary lactate may not add relevant information about occult severe bleeding in non-shocked trauma patients.

## Additional file

Additional file 1: Detailed data of the receiver operating characteristic curves for capillary lactate and shock index to predict significant tranfusion. (DOCX 25 kb)
