# Supplementary material for: Capillary lactate concentration on admission of normotensive trauma patients: a prospective study
Source: Scand J Trauma Resusc Emerg Med. 2016 Jun 7;24:82. doi: 10.1186/s13049-016-0272-x (PMC4896037; doi:10.1186/s13049-016-0272-x)
Supplement: Additional file 1: — Detailed data of the receiver operating characteristic curves for capillary lactate and shock index to predict significant tranfusion. (DOCX 25 kb) [file 13049_2016_272_MOESM1_ESM.docx]

| Receiver operating characteristic (ROC) analysis for capillary lactate concentration on admission to predict a significant transfusion | | | | | |
| --- | --- | --- | --- | --- | --- |
| **Cut-off** | **Sensitivity** | **Specificity** | **Cut-off** | **Sentivity** | **Specificity** |
| >0.95 | 1.00 | 0.01 | >4.75 | 0.50 | 0.64 |
| >1.08 | 1.00 | 0.03 | >4.83 | 0.50 | 0.66 |
| >1.18 | 1.00 | 0.05 | >4.95 | 0.50 | 0.67 |
| >1.25 | 1.00 | 0.09 | >5.08 | 0.50 | 0.68 |
| >1.33 | 1.00 | 0.11 | >5.13 | 0.33 | 0.69 |
| >1.38 | 1.00 | 0.12 | >5.18 | 0.33 | 0.69 |
| >1.48 | 1.00 | 0.15 | >5.3 | 0.33 | 0.71 |
| >1.58 | 1.00 | 0.16 | >5.45 | 0.33 | 0.73 |
| >1.65 | 1.00 | 0.18 | >5.53 | 0.33 | 0.75 |
| >1.73 | 1.00 | 0.19 | >5.58 | 0.33 | 0.76 |
| >1.78 | 1.00 | 0.22 | >5.63 | 0.25 | 0.76 |
| >1.83 | 1.00 | 0.24 | >5.68 | 0.25 | 0.77 |
| >2.03 | 1.00 | 0.25 | >5.8 | 0.25 | 0.78 |
| >2.23 | 1.00 | 0.28 | >6 | 0.25 | 0.79 |
| >2.28 | 1.00 | 0.30 | >6.23 | 0.25 | 0.80 |
| >2.35 | 1.00 | 0.31 | >6.48 | 0.25 | 0.81 |
| >2.43 | 1.00 | 0.33 | >6.65 | 0.25 | 0.81 |
| >2.48 | 1.00 | 0.35 | >6.85 | 0.17 | 0.82 |
| >2.55 | 1.00 | 0.37 | >7.05 | 0.17 | 0.83 |
| >2.63 | 1.00 | 0.40 | >7.13 | 0.08 | 0.84 |
| >2.68 | 1.00 | 0.41 | >7.18 | 0.08 | 0.85 |
| >2.75 | 1.00 | 0.42 | >7.28 | 0.08 | 0.86 |
| >2.83 | 1.00 | 0.43 | >7.5 | 0.08 | 0.87 |
| >2.88 | 1.00 | 0.44 | >7.68 | 0.08 | 0.88 |
| >2.95 | 1.00 | 0.44 | >7.9 | 0.00 | 0.89 |
| >3.05 | 1.00 | 0.45 | >8.15 | 0.00 | 0.90 |
| >3.2 | 1.00 | 0.46 | >8.28 | 0.00 | 0.91 |
| >3.35 | 1.00 | 0.49 | >8.4 | 0.00 | 0.92 |
| >3.43 | 1.00 | 0.52 | >8.63 | 0.00 | 0.93 |
| >3.48 | 1.00 | 0.53 | >8.95 | 0.00 | 0.94 |
| >3.6 | 0.92 | 0.53 | >9.15 | 0.00 | 0.94 |
| >3.73 | 0.83 | 0.54 | >9.45 | 0.00 | 0.95 |
| >3.78 | 0.83 | 0.55 | >9.88 | 0.00 | 0.96 |
| >3.85 | 0.75 | 0.56 | >11.48 | 0.00 | 0.97 |
| >3.93 | 0.75 | 0.57 | >14.35 | 0.00 | 0.98 |
| >4 | 0.75 | 0.58 | >19.88 | 0.00 | 0.99 |
| >4.13 | 0.75 | 0.59 | >4.75 | 0.50 | 0.64 |
| >4.25 | 0.75 | 0.60 | >4.83 | 0.50 | 0.66 |
| >4.35 | 0.67 | 0.61 | >4.95 | 0.50 | 0.67 |
| >4.45 | 0.50 | 0.61 | >5.08 | 0.50 | 0.68 |
| >4.55 | 0.50 | 0.62 | >5.13 | 0.33 | 0.69 |
| >4.65 | 0.50 | 0.63 | >5.18 | 0.33 | 0.69 |

Receiver operating characteristic (ROC) analysis for the pre-hospital shock index to predict a significant transfusion

| **Cut-off** | **Sensitivity** | **Specificity** | **Cutt-off** | **Sentivity** | **Specificity** |
| --- | --- | --- | --- | --- | --- |
| > 0.16 | 1.00 | 0.02 | > 0.94 | 0.36 | 0.81 |
| > 0.35 | 1.00 | 0.03 | > 0.95 | 0.36 | 0.83 |
| > 0.4 | 1.00 | 0.04 | > 0.98 | 0.36 | 0.85 |
| > 0.42 | 0.91 | 0.04 | > 1 | 0.36 | 0.86 |
| > 0.44 | 0.91 | 0.05 | > 1.01 | 0.27 | 0.90 |
| > 0.45 | 0.91 | 0.08 | > 1.03 | 0.27 | 0.91 |
| > 0.46 | 0.91 | 0.10 | > 1.05 | 0.27 | 0.92 |
| > 0.47 | 0.91 | 0.12 | > 1.06 | 0.27 | 0.93 |
| > 0.49 | 0.91 | 0.13 | > 1.07 | 0.27 | 0.94 |
| > 0.51 | 0.91 | 0.18 | > 1.08 | 0.18 | 0.94 |
| > 0.53 | 0.91 | 0.19 | > 1.1 | 0.18 | 0.95 |
| > 0.54 | 0.91 | 0.20 | > 1.14 | 0.18 | 0.96 |
| > 0.55 | 0.91 | 0.23 | > 1.18 | 0.09 | 0.96 |
| > 0.56 | 0.91 | 0.24 | > 1.19 | 0.09 | 0.97 |
| > 0.57 | 0.91 | 0.27 | > 1.32 | 0.00 | 0.98 |
| > 0.58 | 0.91 | 0.29 | > 1.47 | 0.00 | 0.99 |
| > 0.59 | 0.91 | 0.32 | > 0.94 | 0.36 | 0.81 |
| > 0.6 | 0.91 | 0.33 | > 0.95 | 0.36 | 0.83 |
| > 0.61 | 0.91 | 0.34 | > 0.98 | 0.36 | 0.85 |
| > 0.62 | 0.91 | 0.37 | > 1 | 0.36 | 0.86 |
| > 0.63 | 0.91 | 0.38 |  |  |  |
| > 0.65 | 0.82 | 0.41 |  |  |  |
| > 0.66 | 0.82 | 0.42 |  |  |  |
| > 0.67 | 0.82 | 0.43 |  |  |  |
| > 0.68 | 0.82 | 0.47 |  |  |  |
| > 0.69 | 0.82 | 0.49 |  |  |  |
| > 0.71 | 0.82 | 0.55 |  |  |  |
| > 0.73 | 0.73 | 0.56 |  |  |  |
| > 0.74 | 0.73 | 0.59 |  |  |  |
| > 0.75 | 0.73 | 0.60 |  |  |  |
| > 0.76 | 0.64 | 0.62 |  |  |  |
| > 0.77 | 0.64 | 0.64 |  |  |  |
| > 0.78 | 0.55 | 0.66 |  |  |  |
| > 0.8 | 0.55 | 0.67 |  |  |  |
| > 0.81 | 0.55 | 0.69 |  |  |  |
| > 0.82 | 0.55 | 0.71 |  |  |  |
| > 0.84 | 0.45 | 0.74 |  |  |  |
| > 0.86 | 0.45 | 0.75 |  |  |  |
| > 0.87 | 0.36 | 0.75 |  |  |  |
| > 0.89 | 0.36 | 0.77 |  |  |  |
| > 0.9 | 0.36 | 0.78 |  |  |  |
| > 0.92 | 0.36 | 0.79 |  |  |  |
| > 0.93 | 0.36 | 0.80 |  |  |  |
